# Promoting bacterial colonization and biofilm formation for enhanced biodegradation of low-density polyethylene microplastics

**DOI:** 10.1186/s40643-025-00902-8

**Published:** 2025-06-10

**Authors:** Marwa Gamal Eldeen Afify, Ola M. Gomaa, Hussein Abd El Kareem, Mohamed A. Abou Zeid

**Affiliations:** 1 Microbiology Department, National Centre for Radiation Research and Technology (NCRRT), Egyptian Atomic Energy Authority (EAEA), Cairo, Egypt; 2 Faculty of Science, Galala University, Al Galala , Egypt; 3 Microbiology Department, Faculty of Science, Ain Shams University, Cairo, Egypt

**Keywords:** Low-Density polyethylene, Microplastics, Biodegradation, Biofilm formation, Microbial consortium, Gamma radiation

## Abstract

The accumulation of plastic waste presents a significant worldwide environmental challenge. This study aimed to isolate polyethylene-degrading bacteria from marine samples containing plastic waste. Four culturable bacterial isolates: *Micrococcus luteus*,* Bacillus cereus*,* Enterococcus faecalis*,* and Actinomyces* sp. were assessed for their biofilm formation, biosurfactant, and protease production. Gamma irradiation was used to induce structural changes and promote bacterial colonization and biofilm formation on low-density polyethylene microplastics (LDPE MPs). Optimal biofilm formation was achieved in minimal media supplemented with 30% tryptic soy broth, 10% biosurfactant, and 300 µM calcium chloride. The factorial design experiment demonstrated that adding media supplementation significantly improved bacterial colonization and biofilm formation when compared to gamma irradiation. This was supported with Scanning Electron Microscopy (SEM), Energy Dispersive X-ray (EDX) mapping, and Fourier Transform Infrared Spectroscopy (FTIR). The optimized LDPE MP degradation was achieved through a multi-step protocol: (1) samples are pre-treated to 40 kGy gamma irradiation, which resulted in 5.7% Gravimetric weight loss and structural and morphological changes, (2) incubation in biofilm inducing media overnight, and (3) further incubation in minimal media for 30 days. This approach resulted in a total weight loss of 22.5%. In conclusion, synergistic pre-treatment is recommended to promote biofilm and improve biodegradation of LDPE MPs by marine bacteria.

**Supplementary Information:**

The online version contains supplementary material available at 10.1186/s40643-025-00902-8.

## Graphical Abstract

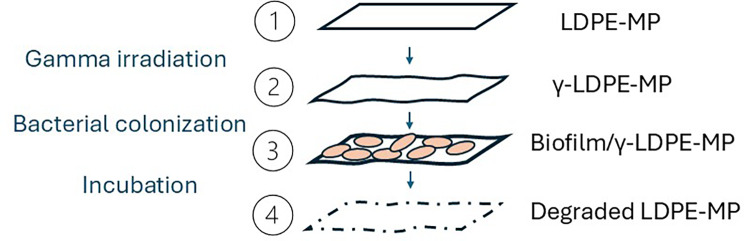

## Introduction

Plastics are among the top harmful xenobiotics due to their synthetic nature, excessive production, and persistence in the environment. About 1.2 billion tonnes of plastics are predicted to enter the environment by 2050 (Kibria et al. 2023). Different types of plastics are used for different purposes, Polyurethane (PUR), Polypropylene (PP), Low Density and High-Density Polyethylene (LDPE, HDPE), Polyvinyl Chloride (PVC), Polyethylene terephthalate (PET) and Polystyrene (PS) (Ru et al. 2020). Low-Density Polyethylene (LDPE) (C_2_H_4_)_n_, is considered among the most common plastics released into the environment, they are used in the manufacturing of toys, squeeze bottles, chemical tank linings, general packaging, heavy-duty sacks, and everyday carrier bags. They are semi-rigid, translucent, waterproof, and low water absorbent (Shivasharana and Kesti 2019). Upon entering the environment, plastics undergo breakdown and fragment into smaller pieces in micro and nanosizes that might not be visible to the naked eye but are more harmful than the bigger macro-sized plastics. Plastic degradation takes place naturally over time through exposure to sunlight (especially ultraviolet radiation), salinity, heat, moisture, and anthropogenic activities (Lin et al. 2022). About 76% of the produced plastic ends up as plastic waste, 14% of which is combusted, 14% recycled and 72% of plastic waste go to landfills. Insufficient plastic recycling and lack of public awareness has led to the displacement of plastic litter from the terrestrial environment and their appearance in aquatic ecosystem where extensive fragmentation to micro (50 μm and 5 mm) and nanosized particles (less than 100 nm particle size) takes place. This leads to more hazardous effects as marine creatures ingest fragmented plastics (Dimassi et al. 2022). In soil, it inhibits the growth of earthworms, decreases soil pH, and affects seed germination causing a reduction of plant shoots (Boots et al. 2019). Eventually, microplastics reach the food chain and were even reported to appear in human blood (Leslie et al. 2022). MPs present in the human tissues are considered foreign bodies and were reported to trigger immunoreactions. MPs present in human placenta may be responsible for changes in key receptors regulating maternal-foetal communication and transfer of immune cells from mother to foetus and may also result in foetal growth restrictions and preeclampsia (Sridhar et al. 2024). Realizing the plastic waste problem has prompted research to find ways to degrade plastic waste. Pyrolysis of microplastics was performed at 450^o^C for complete removal from sewage sludge (Ni et al. 2020). Photocatalytic degradation of polystyrene microplastics (in the presence of polyaluminium chloride as a coagulant) was reported using ultraviolet and titanium dioxide (Meera et al. 2023), UV irradiation step took 45 days. Water ozonation using O_3_ and O_3_/H_2_O_2_ resulted in a 1.3 to 26.7% loss of surface area that affected the chemical and physical characteristics of five different microplastics (Ziembowicz and Kida 2024). Biodegradation has long been considered a safe, no-energy degradation process that can turn MPs into simple organic compounds (Krueger et al. 2015). Biodegradation of microplastics is categorized into biodeterioration, biofragmentation, assimilation, and mineralization (Nawaz 2020). Different Genus, such as *Bacillus*, *Pseudomonas*, *Cyanobacteria*, *Penicillium*, *Aspergillus*, and different microalgae showed remarkable MP biodegradation (Sridhar et al. 2024). The media used for plastic biodegradation differs, e.g. defined media, soil, sweater, and landfill leakage water (Krueger et al. 2015). During biodegradation, microbes form biofilm on MPs which leads to pitting that cracks the MP surface facilitating their attack by microbial enzymes. The extracellular MP biodegradation mechanism starts with bacterial attachment, colonization, biofilm formation, and enzyme secretion, which leads to the deterioration and fragmentation of MPs. (Wang et al. 2024). Biofilm formation took around 7 days for marine bacteria to colonize LDPE (Harrison et al. 2011) and took 40 days to develop *Pseudomonas* sp. biofilm on LDPE films (Kyaw et al. 2012). Therefore, some studies resorted to synergistic biodegradation approaches to induce physical or chemical changes in MPs that would facilitate the biodegradation process. For example, Tween 20 was used as a pretreatment step for polyethylene terephthalate before using yeast-assisted degradation (Giyahchi and Moghimi 2024). High temperature, nitric acid, and ionizing radiation were used as a pre-treatment for LDPE sheets before biodegradation using *Aspergillus fumigatus* and *Aspergillus carbonarius* (El-Sayed et al. 2021). Ionizing radiation is one of the effective plastic pre-treatment approaches. The high energy of ionizing radiation results in break of the covalent polymer backbone and generation of free radicals which result in random chain scission and the formation of oxidized groups such as carboxyl, hydroxyl and carbonyl (Ponomarev et al. 2022). This reduces the polymer molecular weight, mechanical properties and changes the surface of plastics from hydrophobic to hydrophilic which makes it easy for bacteria to colonize and form biofilm. Biofilm formation is the step preceding enzymatic degradation of the polymer. Therefore, both pre-treatment and biofilm formation are considered crucial pre-treatment steps for the enhancement of MP microplastic biodegradation. From this standpoint, the present work aimed to isolate marine bacteria for the biodegradation of Low-Density Polyethylene microplastics and enhance bacterial colonization and biofilm formation using gamma radiation and media supplementation to leverage the biodegradation process.

## Materials and methods

### Bacterial isolation and identification

Bacteria species were isolated from discarded plastic buried in wet sand and seawater taken from the Egyptian North coast (260 Km from Cairo). About 10 mL of the sample was mixed with 90 mL of normal saline water (0.9% NaCl). The mixture was shaken for 2 h at 150 rpm in an orbital shaker at 30^o^C. The resulting suspension was subjected to 5X serial dilution. About 0.1 mL dilutions were dispensed on freshly prepared Tryptic Soy Agar (TSA) under aseptic conditions. TSA (37 g/L) was prepared as indicated by the manufacturer, 20 g/L agar was added and autoclaved at 121^o^C for 20 min under pressure. The inoculated media plates were incubated at 30^o^C for 24 h. Developed colonies were further sub-cultured on freshly prepared (TSA) in triplicates to obtain individual pure cultures and ensure the purity of the samples prior to identification. Purified isolates were stained using the Gram-stain procedure which is a differential staining procedure that involves adding different stains to bacteria to make them visible to distinguish between Gram-positive and Gram-negative bacteria. Isolates were identified using the VITEK 2 Compact equipment and VITEK 2 SYSTEM software (bioMérieux Inc, USA). GP ID REF21341 (identification-Gram-positive bacteria) cards were referred to for identification. All the test procedures were followed according to the manufacturer´s instructions . Identification was confirmed using Matrix Assisted Desorption Ionization-Time of Flight (MALDI-TOF). A single colony was smeared on stainless-steel plate, 1µL was added to each sample, 1 µl of α-Cyano-4-hydroxycinnamic acid (HCCA) matrix dissolved in 50% HPLC water, 47.5% ACN, 2.5% TFA and water, was aliquoted on each spot after being dried at room temperature. Mass spectra were obtained using linear mode MALDI-TOF (SAI) LT3, UK operating in linear mode and extracting positive ions with an accelerating voltage of 25 kV. Both genus and species of isolated bacteria were scored based on spectrum comparison to those available in the MALDI-TOF library.

### Polyethylene microplastics

The PE microplastics were prepared by cutting used PE plastic single-use bags. The same batch was used throughout the study. The plastic samples were cut using a scalpel and scissors into (2 × 2 mm) sizes to obtain equal microsize films. The microplastic films were washed with 70% ethanol and oven-dried at 50^o^C overnight or until sample dryness.

### LDPE microplastic exposure to different gamma radiation doses

Gamma radiation was performed using the Co^60^ gamma Canadian unit (The National Centre for Radiation Research and Technology - NCRRT), Nasr City, Cairo-Egypt. The plastic films were subjected to different gamma radiation doses, namely: 10, 20, 40, and 80 kGy. The dose rate at the time of the experiment was 0.9 kGy/h for dry irradiation.

### Biodegradation of LDPE microplastics

Prior to the experiment, the PE microplastic samples were disinfected with ethanol 70% (30 min soaking) and air-dried in the laminar airflow chamber. For the assessment of growth and biodegradation, Minimal Media broth medium consisting of the following in g/L; MgSO_4_ 0.2, CaCl_2_ 0.02, K_2_HPO_4_ 1.0, KH_2_PO_4_ 1.0, NH_4_NO_3_ 1.0 and FeCl_3_ 0.05 was used throughout the entire experiment. No carbon source was supplemented to the medium to eliminate the strains’ growth reliance on the supplementary carbon. The medium pH was adjusted to 7.0 ± 0.2 at 25^o^C before sterilization. The assay was evaluated by inoculating 10% of the bacterial culture (OD_600_ = 1.0) into liquid culture (50 mL of Minimal Media with 50 mg of PE microplastics in 250 mL conical flask). Flasks were incubated at 30^o^C and test samples were taken periodically to assess the changes. The uninoculated Minimal Medium with PE microplastics was used as the negative control. All experiments were performed in triplicate.

### Gravimetric analysis of residual PE microplastics

The remaining PE microplastics were retrieved from the medium through filtration, sequentially washed with 70% ethanol solution and 2% Sodium Dodecyl sulphate (SDS) for maximum removal of any formed biofilm and were left to dry overnight, and the polymer final weight was measured using the analytical balance. The weight loss percentage of PE microplastics was calculated using the following equation:

Weight loss (%) = (Wo − W)/Wo) × 100.

Where: Wo is the initial weight of the PE microplastics (g) and W is the final weight of the PE microplastic (g).

### Fourier transform infrared (FTIR) analysis of PE microplastics

Infrared spectroscopic analyses were performed on irradiated PE plastic films to identify the main functional groups. The normalised intensity of the functional groups was determined based on the transmittance (%). Analyses were conducted on both PE microplastic samples and on the uninoculated control PE microplastic samples. Scanning was performed from 400 to 4000 cm^− 1^ using FTIR, BRUKER VERTEX 70 device at NCRRT. The resulting analytical spectrum was analysed in comparison to references to identify the functional groups.

### Thermogravimetric analysis (TGA)

The TGA of the PE microplastics was performed using TGA-50 Schimadzu (Shimadzu Scientific Instruments, Inc., Japan). All samples were heated from room temperature to 600 ^o^C at a heating rate of 10 ^o^C min^− 1^ in the presence of nitrogen flow of 10 mL/min.

### Scanning Electron microscopy (SEM) and energy dispersive X-ray (EDX) mapping

Scanning Electron Microscopy images were captured using a Zeiss evo15 SEM (Germany). The MPs were placed on brass stubs using double-sided adhesive tape before coating with a thin layer of gold under reduced pressure. The images were captured at 3000 X magnifications using an electron beam high voltage of 20 kV. Energy Dispersive X-Ray (EDX) mapping was used for elemental mapping of PE, irradiated PE, irradiated PE with biofilm, and irradiated PE with promoted biofilm, the elements chosen were C, O, N, P, and S.

### Characterization of LDPE biodegrading bacteria

Different assays were performed to depict the pathway of biodegradation of LDPE by each isolated bacterium. Each bacterium was used to inoculate a 250 mL Erlenmeyer flask containing a working volume of 100 mL glycerol supplemented nutrient broth (GSNB) media which contained in g/L Meat Extract 1, Yeast Extract 2, Peptone 5, NaCl 5, Glycerol 30 mL. The cultures were incubated for 72 h at 37^o^C and 150 rpm. At the end of the incubation period, culture filtrates were tested by drop collapse method and emulsification index (E24%) to test if biosurfactants were produced by the isolates. Congo red Brain Heart Infusion (BHI) media was used to identify biofilm-forming bacteria. BHI broth (37 g/L) supplemented with sucrose (50 g/L), agar (20 g/L), and Congo red dye (0.8 g/L) was used for the CRA method (Freeman et al. 1989). Protease detection was performed by observing clear zones in skimmed milk agar media plates.

### Optimization of bacterial colonization and biofilm formation on PE microplastics

To enhance biofilm colonization, three additives were added to the mineral media (as the base media) in different concentrations to mineral media: (1) TSB media was added as 0, 5, 10, 20, and 30%. (2) Biosurfactants produced at the end of incubation in GSNB media were added as 0, 5, 10, 20, and 30%. (3) CaCl_2_ solution was added at a final concentration of 0, 150, 300, 600, and 900 mM. The final OD was obtained after 24 h using a UV-visible spectrophotometer (SPECORD 210 plus, analytic Jena). The biofilm was visualized using the crystal violet method. The results were scored at the end of each experiment and plotted on Excel, R^2^ value was calculated from the linear plot for TSB, biosurfactant, and CaCl_2_ individually.

### Factorial design

To identify the effect of gamma radiation and the addition of other supplements to promote bacterial colonization and biofilm formation, Placket Burman design was performed using Minitab 20. Factors chosen were gamma radiation and supplements 2^2^ (-1, + 1) with 12 runs. Gamma radiation doses chosen were 0 and 40 kGy, while the supplement mixtures were absent or present, a mixture of 30% v/v of TSB, 10% biosurfactant, and 300 µM CaCl_2_ which were chosen based on the previous experiment. The obtained results were plotted as a Pareto chart and Main Effects Plot for biofilm response and the R^2^ was represented in the Model summary equation.

Model summary

$${{S\,\,\,R - sq\,\,R - sq(adj)\,R - sq(pred)} \over {8.16043\,\,95.74\% \,\,\,\,\,94.80\% \,\,\,\,92.43\% }}$$

### Statistical analysis

Where applicable, statistical analysis was calculated using Minitab 20 and the results were plotted as SE.

## Results and discussion

### 1-Isolation and identification of PE degrading strains isolated from a marine sample

**Fig. 1:**
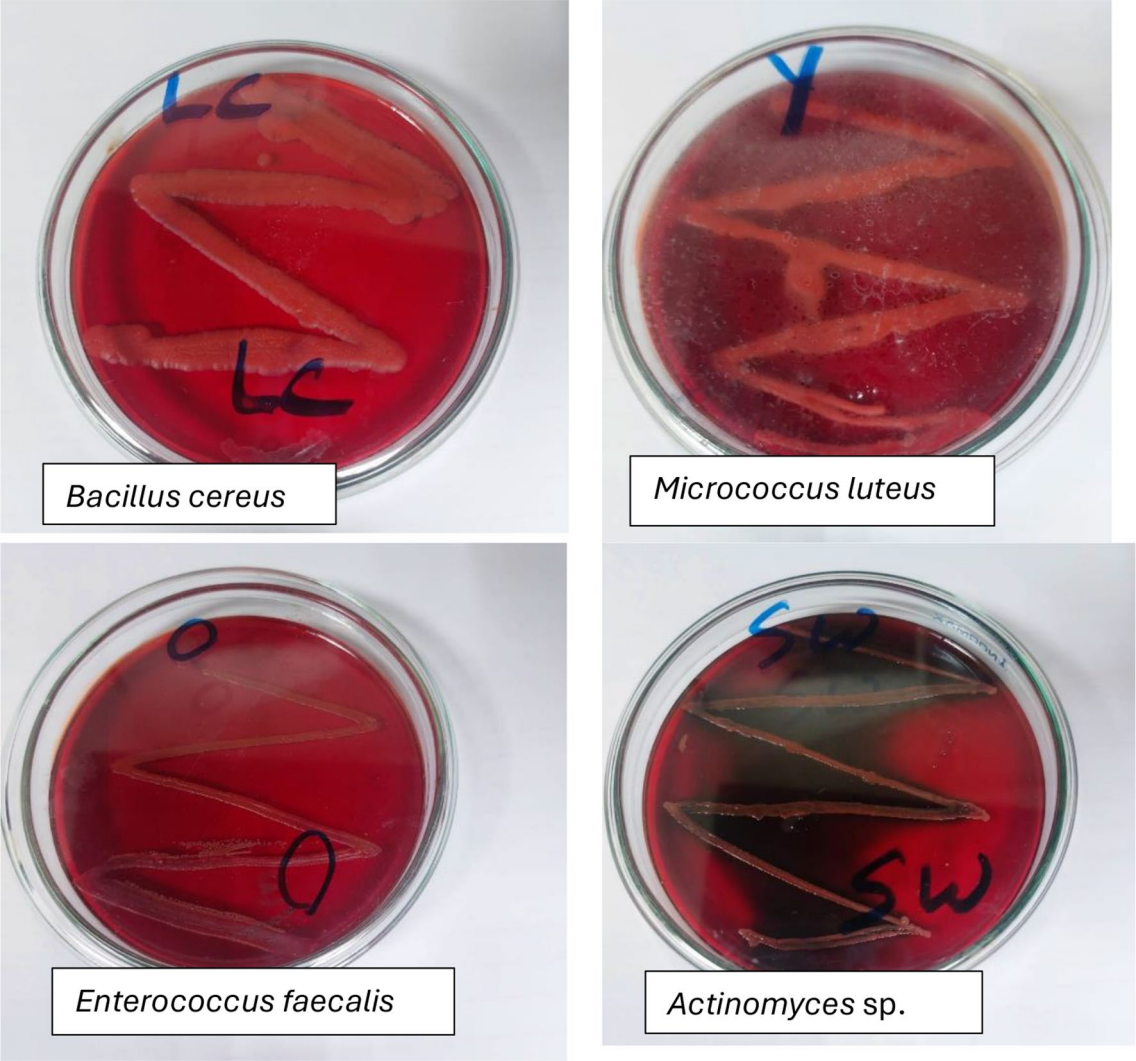
Biofilm formation of each isolate and the mixture of marine bacteria on CRA plates

Four culturable isolates were identified using Vitek and were confirmed using MALDI-TOF MS as *Bacillus cereus* with a score of 0.92, *Entaerococcus faecalis* with a score of 0.9, *Micrococcus luteus* with a score of 0.98 and *Actinomyces marimammilians* with a score of 0.82 (Table 1). Each strain was tested for possible characteristics that can be that maybe involved in microplastic degradation. Table 2 shows that *Bacillus cereus* and *Micrococcus luteus* form biofilm, produces biosurfactant and they both show positive result for protease and catalase enzymes. *Entaerococcus faecalis* forms biofilm but doesn’t produce biosurfactant or assayed enzymes, while *Actinomyces marimammilians* doesn’t form biofilm nor produce biosurfactants or assayed enzymes. *Bacillus cereus*, *Micrococcus luteus* and *Entaerococcus* sp. were previously reported among LDPE degrading bacteria (Nowak et al. 2011; Sen & Raut 2015, Wang et al. 2023). There are five morphotypes, the closest description of *Micrococcus luteus* and *Entaerococcus faecalis* colonies on CRA can be described as sbam (smooth, brown, and mucoid). Sbam are characterized as lacking cellulose synthesis but overproducing capsular polysaccharide (Merino et al. 2019). Table 2; Fig. 1 confirm that each of the bacterial isolates has its characteristics, it is expected that the use of bacterial consortium will result in enhancing bacterial colonization, biofilm formation, and biodegradation mechanism of microplastics as seen later by gravimetric and chemical analyses of LDPE MPs. The biodegradation of LDPE is complex and not fully understood (Jin et al. 2023), with two different strategies reported in the literature to elucidate the potential mechanisms. The first approach studies single-strain degradation using pure strains able to degrade LDPE, that approach is a convenient way to investigate metabolic pathways or to evaluate the effect of different environmental conditions on LDPE degradation. However, a disadvantage of this approach is that it ignores the possibility that LDPE biodegradation can result from a cooperative process between different species. Such limitations are avoided by the second approach, in which the use of complex environments and microbial communities are applied (Sen & Raut 2015). Therefore, in the present work, the bacterial consortium was used for the upcoming experiments based on the individual characteristics of the isolated strains.

**Table 1:** MALDI-TOF Identification of different bacteria isolates from marine sea/sand sample

	Isolate Acronym	Isolate name	Gram stain	Score
1	*LC*	*Bacillus cereus*	+ve	0.92
2	*SW*	*Entaerococcus faecalis*	+ve	0.9
3	*Y*	*Micrococcus luteus*	+ve	0.98
4	*O*	*Actinomyces marimammilians*	+ve	0.82

**Table 2:** Assay of some biological parameters to depict the LPDE microplastic biodegradation by marine bacteria

Microorganism	Biofilm	Biosurfactant(E%24)	Protease	Catalase
*Actinomyces marimamilian*	-ve	-ve	-ve	-ve
*Micrococcus luteus*	+ve	50%	+ve	+ve
*Bacillus cereus*	+ve	60%	+ve	+ve
*Enterococcus faecalis*	-ve	-ve	-ve	-ve
Consortium	+ve	45%	+ve	+ve

### 2-Enhancing bacterial colonization and biofilm formation

**Fig. 2:**
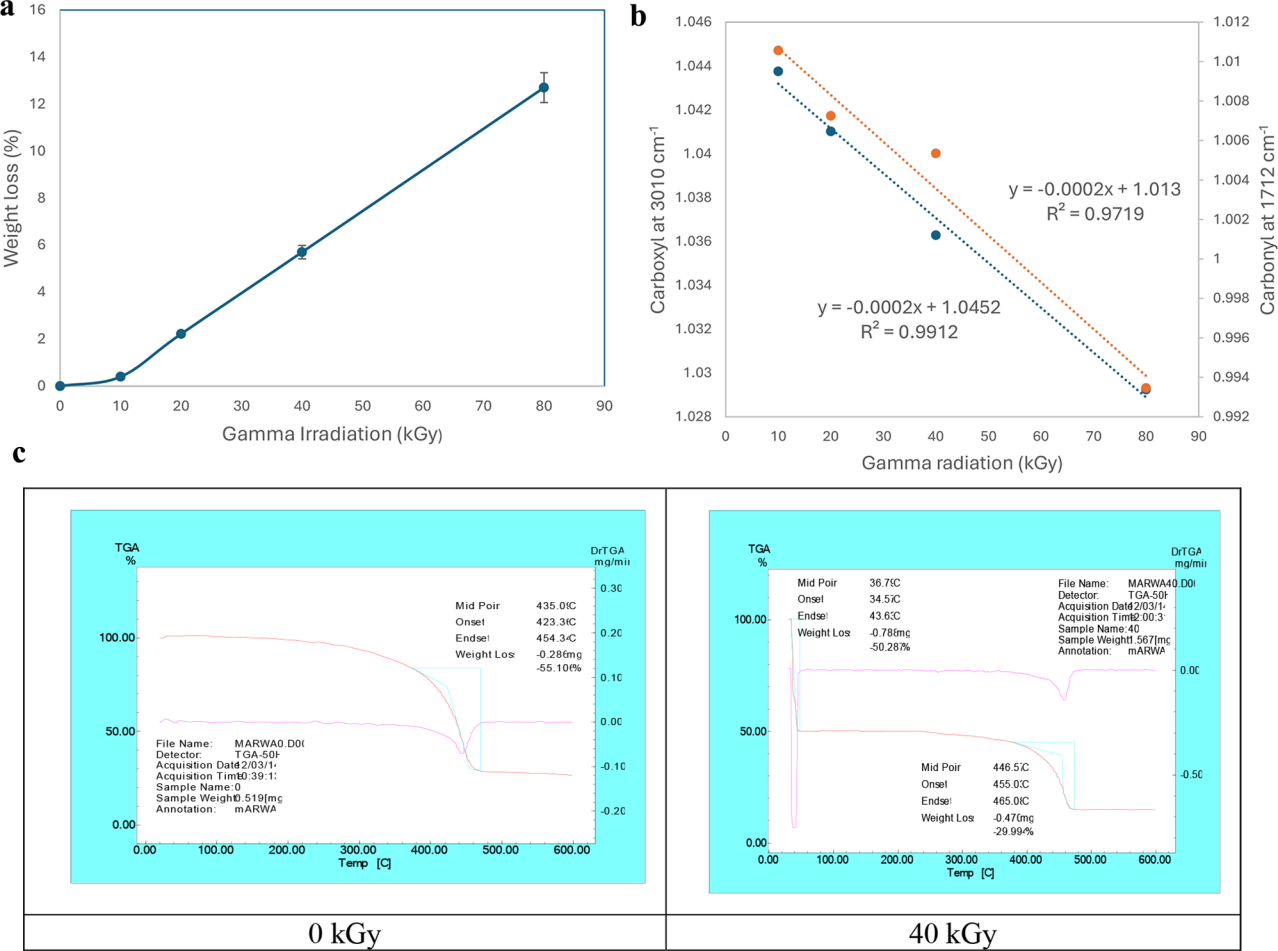
(**a**) Gravimetric results for microplastic exposed to gamma radiation at 0, 10, 20, 40 and 80 kGy as pre-treatment. (**b**) Transmittance (%) at 3010 and1712 cm^− 1^ FTIR for microplastic exposed to 0, 10, 20, 40 and 80 kGy gamma irradiation as pre-treatment, (**c**) TGA results for microplastic exposed to 0 and 40 kGy gamma irradiation as pre-treatment

**Fig. 3:**
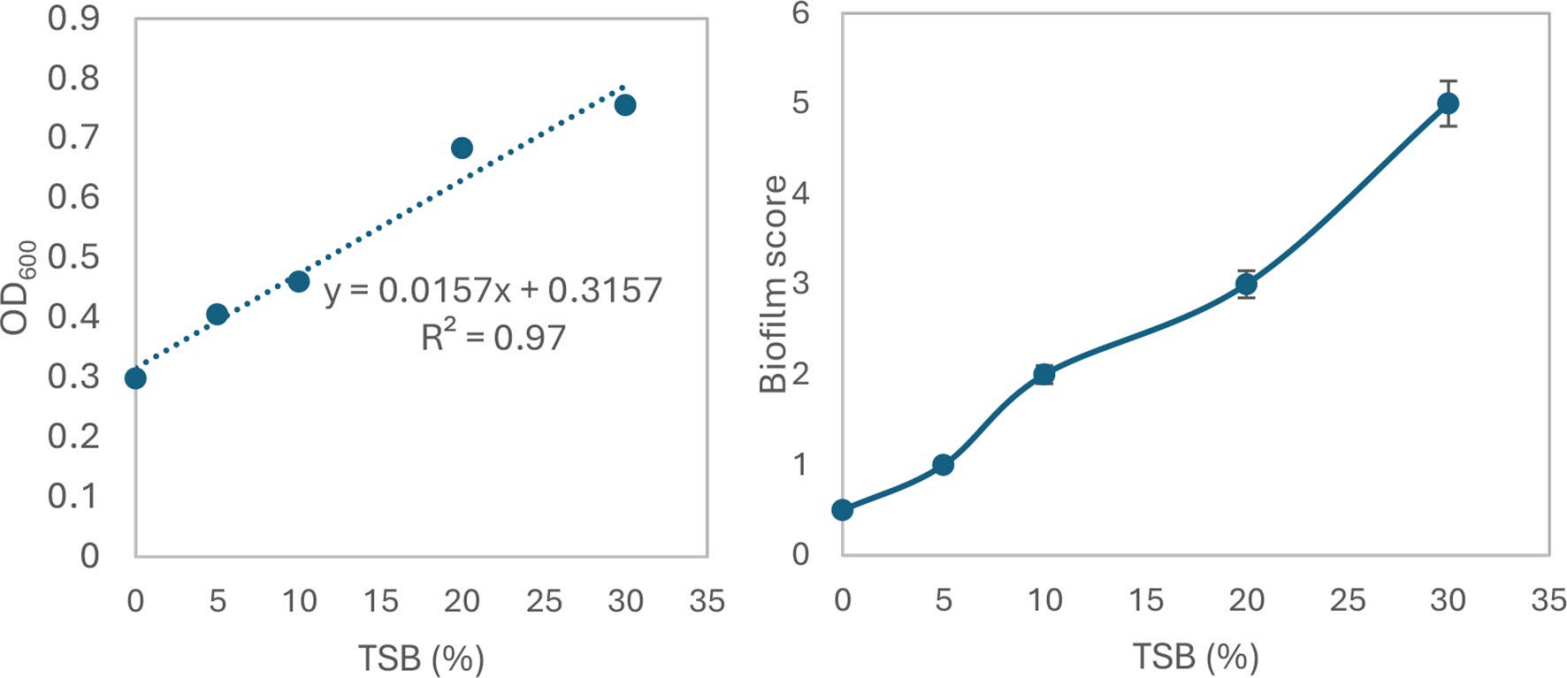
Effect of TSB on the growth of bacterial consortium and biofilm formation using CV method

**Fig. 4:**
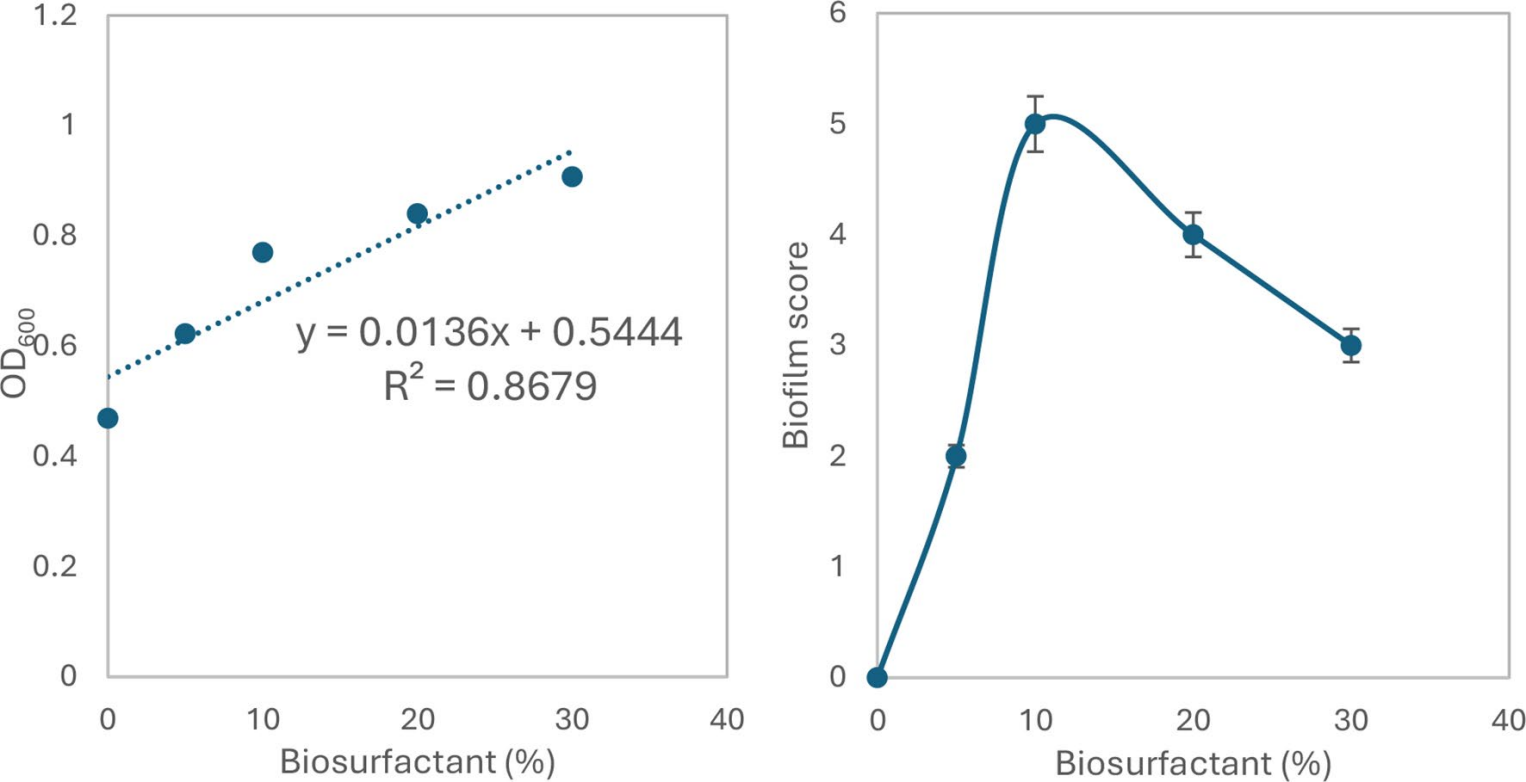
Effect of Biosurfactant on growth of bacterial consortium and biofilm formation using CV method

**Fig. 5:**
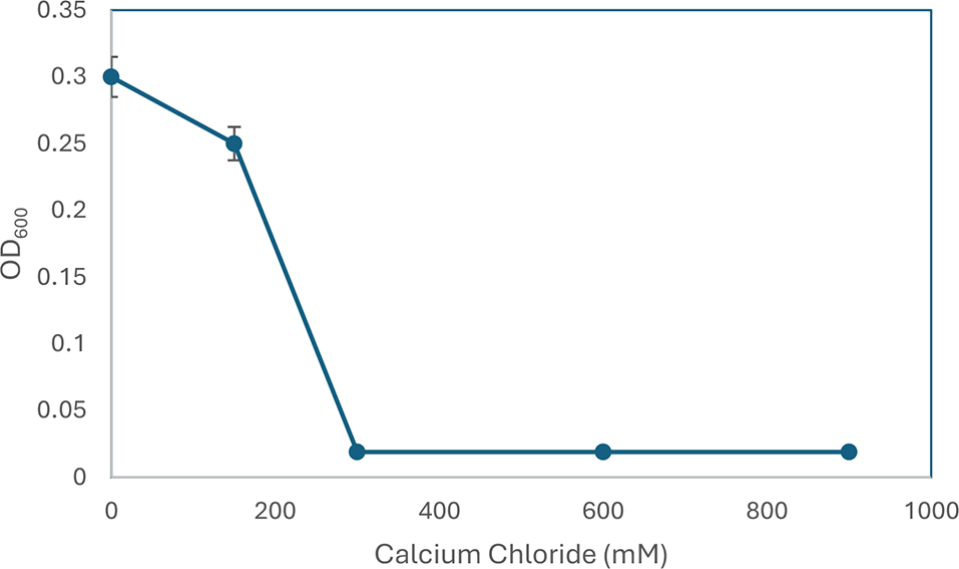
Effect of CaCl_2_ on settling of bacterial cells

**Fig. 6:**
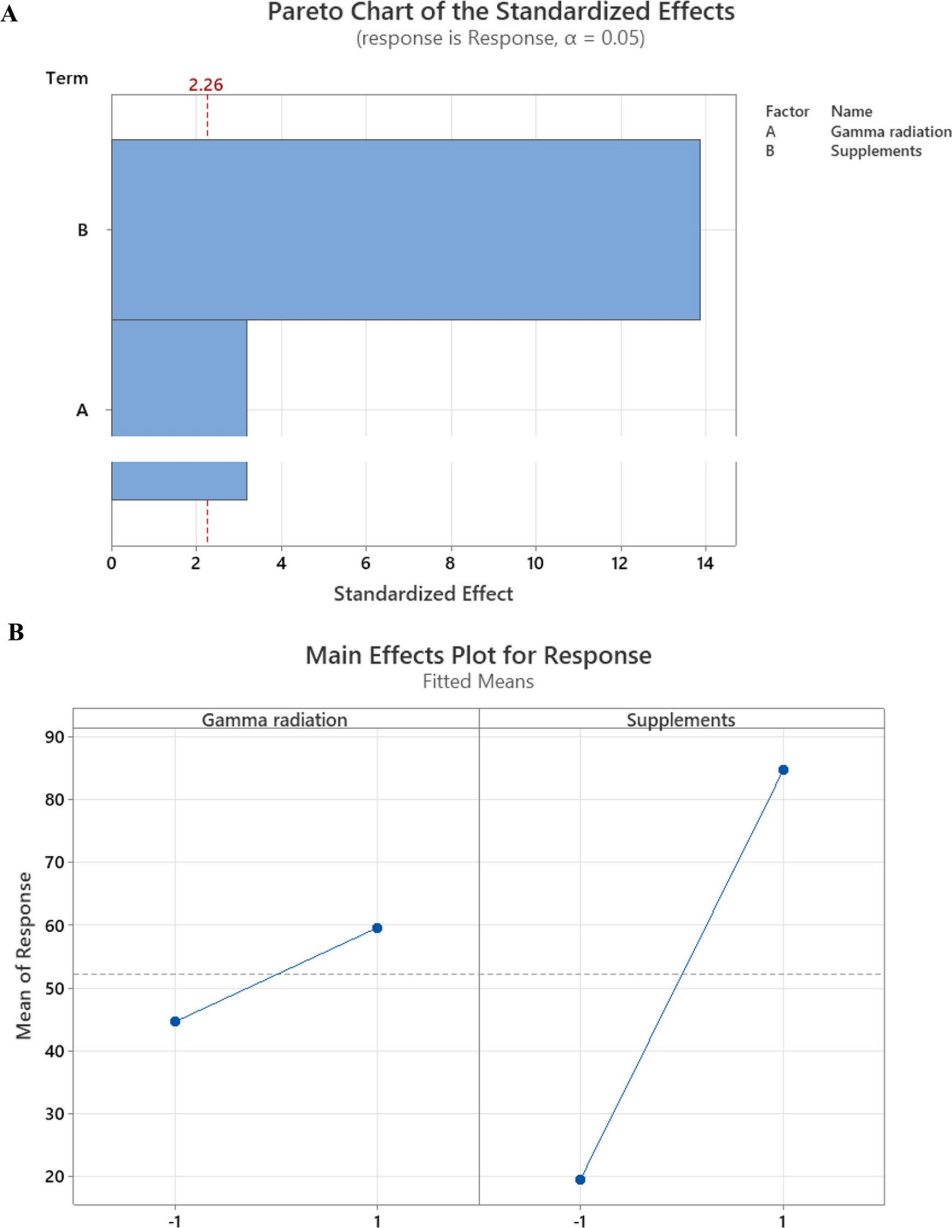
(**a**) Preto chart for gamma irradiation and supplementation for bacterial colonization and biofilm formation. (**b**) Main effect chart for gamma irradiation and supplementation for bacterial colonization and biofilm formation

**Fig. 7:**
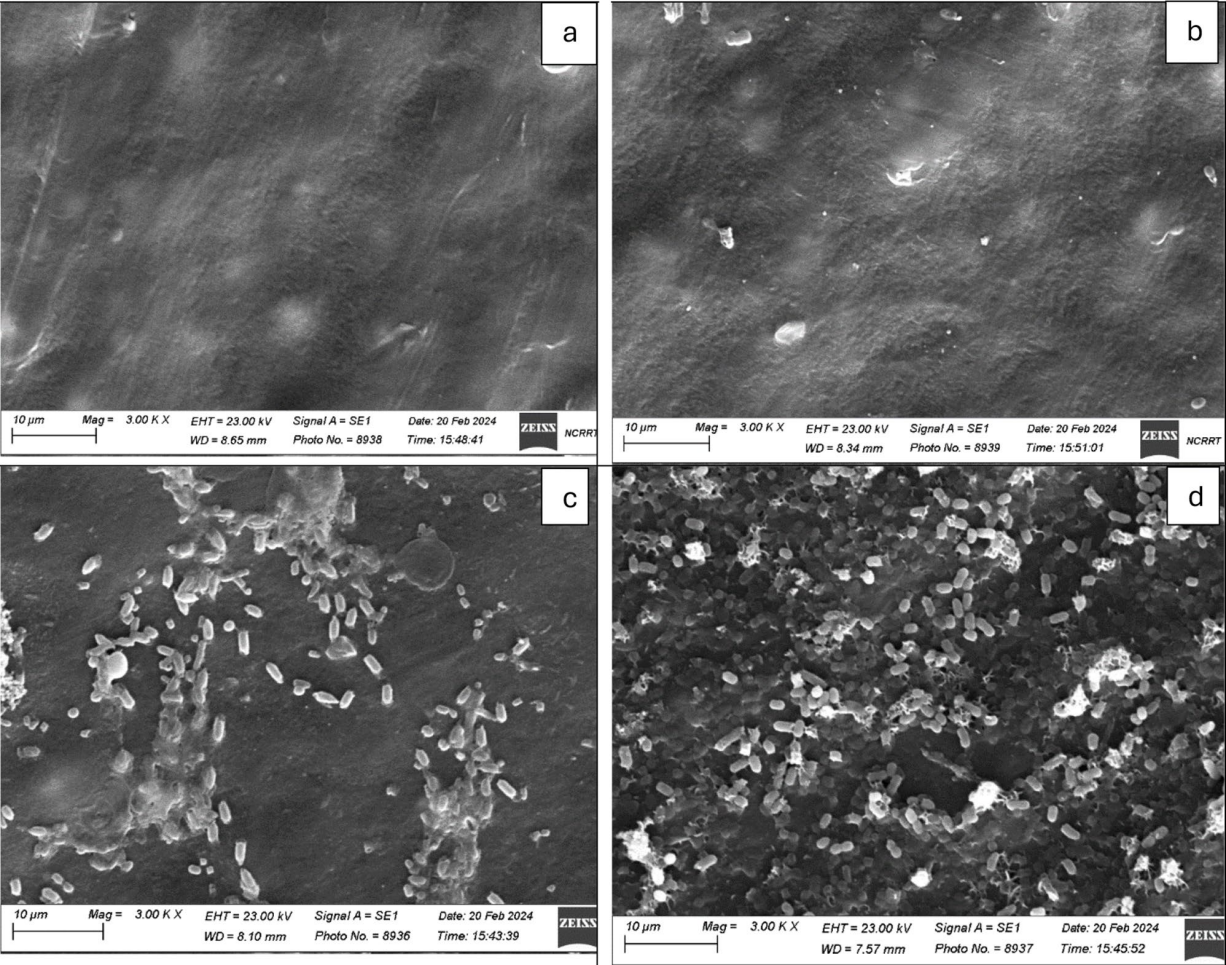
SEM images for LDPE MPs (**a**), Gamma irradiated LDPE MPs (**b**), Biofilm formed on LDPE MPs (**c**) and enhanced biofilm after adding supplements to gamma irradiated LDPE MPs (**d**). Magnification at 3000 x

**Fig. 8:**
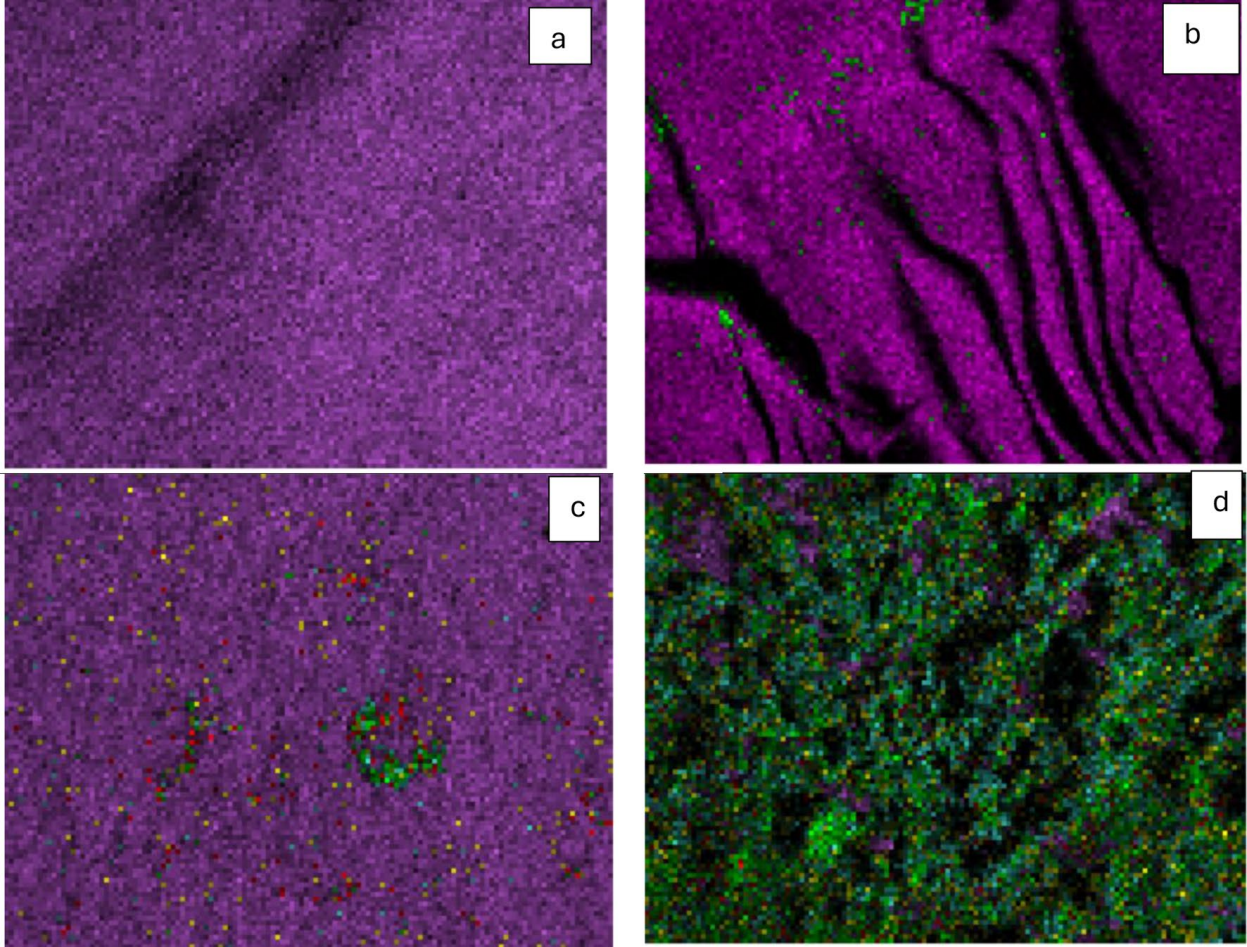
(**a**) EDX mapping representing LDPE MPs (**a**), Gamma irradiated LDPE MPs (**b**), 24 h biofilm formed on LDPE MPs (**c**) and 24 h biofilm after adding supplements to gamma irradiated LDPE MPs (**d**). Purple represents carbon, dark green represents Oxygen, light green represents phosphorus, red represents nitrogen and yellow represents sulphur. Spectrum and tables for the elemental analysis are in S1

**Fig. 9:**
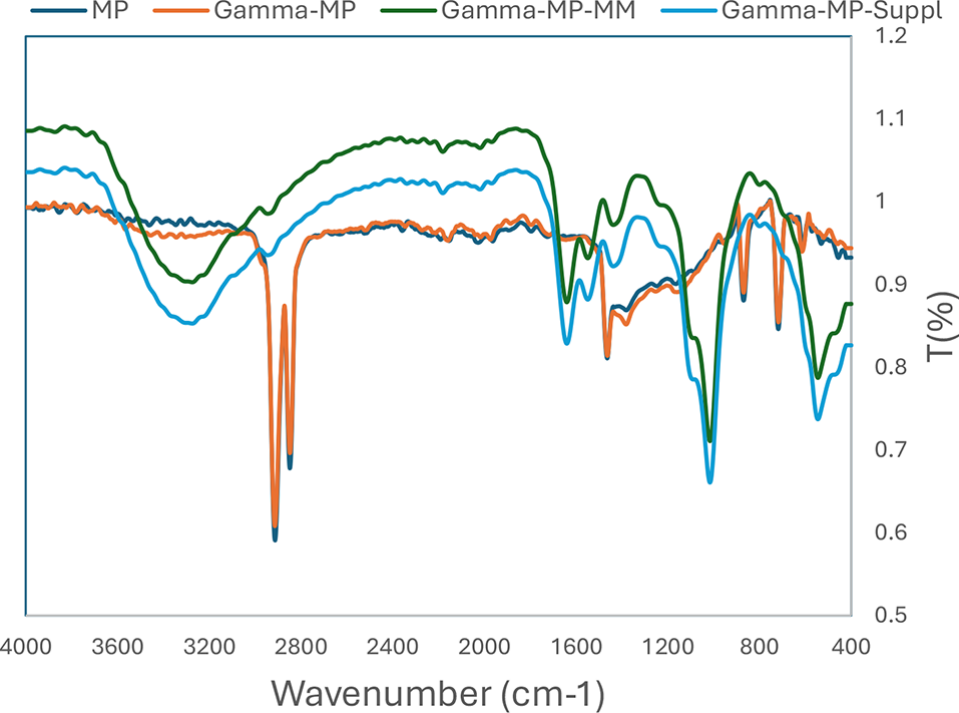
FTIR spectra for LDPE. LDPE gamma irradiated, 24 h biofilm grown on irradiated LDPE and 24 h biofilm supplemented and grown on irradiated LDPE

Bacterial colonization is an important prerequisite for biodegradation since LDPE is hydrophobic, which makes it difficult for bacteria to attach to it. To enhance bacterial colonization and biofilm formation, there were two approaches: (1) exposing MPs to gamma radiation, and (2) adding supplements to the minimal media to accelerate the biofilm process. Gravimetric analysis results showed that the weight loss increased with exposure to increasing doses of gamma irradiation (Fig. 2a). The weight loss reached 5.7% and 12.7 after exposure to doses of 40 kGy and 80 kGy. Two distinctive FTIR peaks represent the effect of gamma irradiation on LDPE MPs. Figure 2b demonstrates the reduction of Transmittance (%) at 3010 cm^− 1^ which represents OH stretching carboxylic acid and at 1710 cm^− 1^ which represents C = O ester for the carbonyl group. The obtained results show that R^2^ were 0.97 and 0.99which reflects an oxidation process taking place upon the exposure to gamma irradiation. Since the Gamma irradiation effect was effective at 40 kGy and as it takes time for higher doses, in addition to cost reasons, 40 kGy was chosen as the pre-treatment dose for the upcoming experiments. A further confirmation was achieved through Thermal Gravimetric Analysis (TGA) assessed before and after exposure to the selected 40 kGy gamma radiation. Results show a weight loss of -0.286 mg for the untreated sample and of -0.788 mg for the gamma irradiated sample (Fig. 2c). Biofilm formation was optimized for the mixed bacterial consortium when adding different supplements to minimal base media. The results reflect an increase in biofilm formation that reached its maximum when adding 30% TSB to minimal media that was concomitant to bacterial cell growth (Fig. 3). A 10% added biosurfactant to minimal media was optimal for biofilm formation which decreased at higher concentrations despite the increase recorded in bacterial cell growth (Fig. 4). Bacterial cells showed biosettling upon the addition of 300mM calcium chloride to minimal media (Fig. 5). To identify the key contributor to biofilm formation (gamma radiation or adding supplements to the media), factorial design was performed. and the pareto chart and main effect plots show that although both contribute to biofilm formation, adding supplements to minimal media plays a key role since it involves bacterial growth and metabolite effects such as biosurfactant (Figs. 6a&b). The model summary shows R^2^ of 95.74%. Gamma radiation provides enough pits that facilitate the attachment of bacteria on LDPE MPs surface which is the initial step in biofilm formation. On the other hand, the addition of supplements to the minimal media promoted colonization, TSA is considered important in enhancing microbial biofilm formation due to its components (Lade et al. 2019). Biosurfactants play several roles that enhance biofilm formation, reduce hydrophobicity, and control cell motility as well as cell-to-cell communication known as quorum sensing (Sharma et al. 2021). Optimizing the concentration of biosurfactant added to the culture media is very important. It can enhance biofilm formation by increasing exopolysaccharide production, however, increasing the concentration may inhibit biofilm formation or even affect cell viability (Gomaa et al. 2021). The addition of calcium chloride induces cell signalling in bacteria and boosts biofilm formation (Liu et al. 2020), calcium chloride has also been reported to induce exopolysaccharide production (Gomaa et al. 2022). To confirm this, SEM, EDX mapping, and FTIR were applied for untreated, gamma-irradiated LDPE MPs and biofilm formation on LDPE MPs before and after adding supplementation. The results show that exposing LPDE microplastics to different doses of gamma radiation resulted in changes in the physical and chemical nature of LDPE. SEM images were analysed to visualize the surface of LDPE MPs before and after exposure to gamma radiation. Images show changes in the surface of LDPE MPs with evident perforations or small holes on the surface for 40 kGy exposed MPs as compared to non-irradiated samples (Fig. 7a&b). Environmental factors such as pH, salinity, temperature, and light affect the surfaces of plastics and cause holes, grooves, cracks, and disintegration (Dimassi et al. 2022). The changes induced results in the increase in biofilm formation as seen in Fig. 7c. The captured images show that biofilm formation was accelerated upon the addition of supplements (Fig. 7d). EDX mapping shows the main component in PE which is Carbon (Fig. 8a), Oxygen appeared upon gamma irradiation which confirms oxidation upon exposure to 40 kGy (Fig. 8b). Biofilm formation was confirmed based on the appearance of Nitrogen, Phosphorous, and Sulphur (Fig. 8c), in addition, biofilm formation increased upon adding media supplements to the media and this was confirmed by the increase in O, P, S, and N in comparison to C (Fig. 8d). Spectra for EDX mapping with elemental analysis confirm the above results and are available as supplementary material (S1). To estimate structural changes for LPDE MPs before and after gamma radiation, FTIR spectrum was obtained (Fig. 9). The results show that non-irradiated LDPE shows characteristic peaks at 2920.7 cm^− 1^ and 2851.2 cm^− 1^ both characteristic for C-H alkane, 1463 cm^− 1^ characteristic for C-H bending, 869.81 cm^− 1^ characteristic for C-H bending, 717.45 cm^− 1^ characteristic for C-C bending and 624.87 cm^− 1^ characteristic for C-Cl. Exposing LDPE MPs to 40 kGy resulted in the appearance of peaks at 1380.9 cm^− 1^ characteristic for C-O-H bending mode, 1178.39 cm^− 1^ and 1006.74 cm^− 1^ both characteristic for C-O stretching mode, and 960 cm^− 1^ characteristic for = C-H out-of-plane bending mode. Biofilm formation was confirmed by the appearance of peaks at 3276.75 broad peak characteristic for O-H carboxylic acid, 2927.67 cm^− 1^ characteristic for C-H alkane, 2177.43 characteristic for C = C = O, 1639.34 characteristic for C = C stretching alkene, 1544.83 characteristic for N-O, 1427.19 characteristic for O-H carboxylic acid, 1205.39 characteristic for C-N stretching amine, 1062.67 characteristic for C-O primary alcohol and 541.94 cm^− 1^ characteristic for C-Cl for both biofilms formed after 24 h growth in minimal media and that grown in minimal media supplemented with TSA media, biosurfactant and calcium chloride. The only change is the decrease in the transmittance of the spectrum for the latter which suggests more biofilm formation and more transparency of LDPE MPs. The results obtained are in accordance with that obtained by El-Sayed et al. (2021) and Liu et al. (2022).

### 3- LDPE MPs biodegradation using marine microbial consortium

**Fig. 10:**
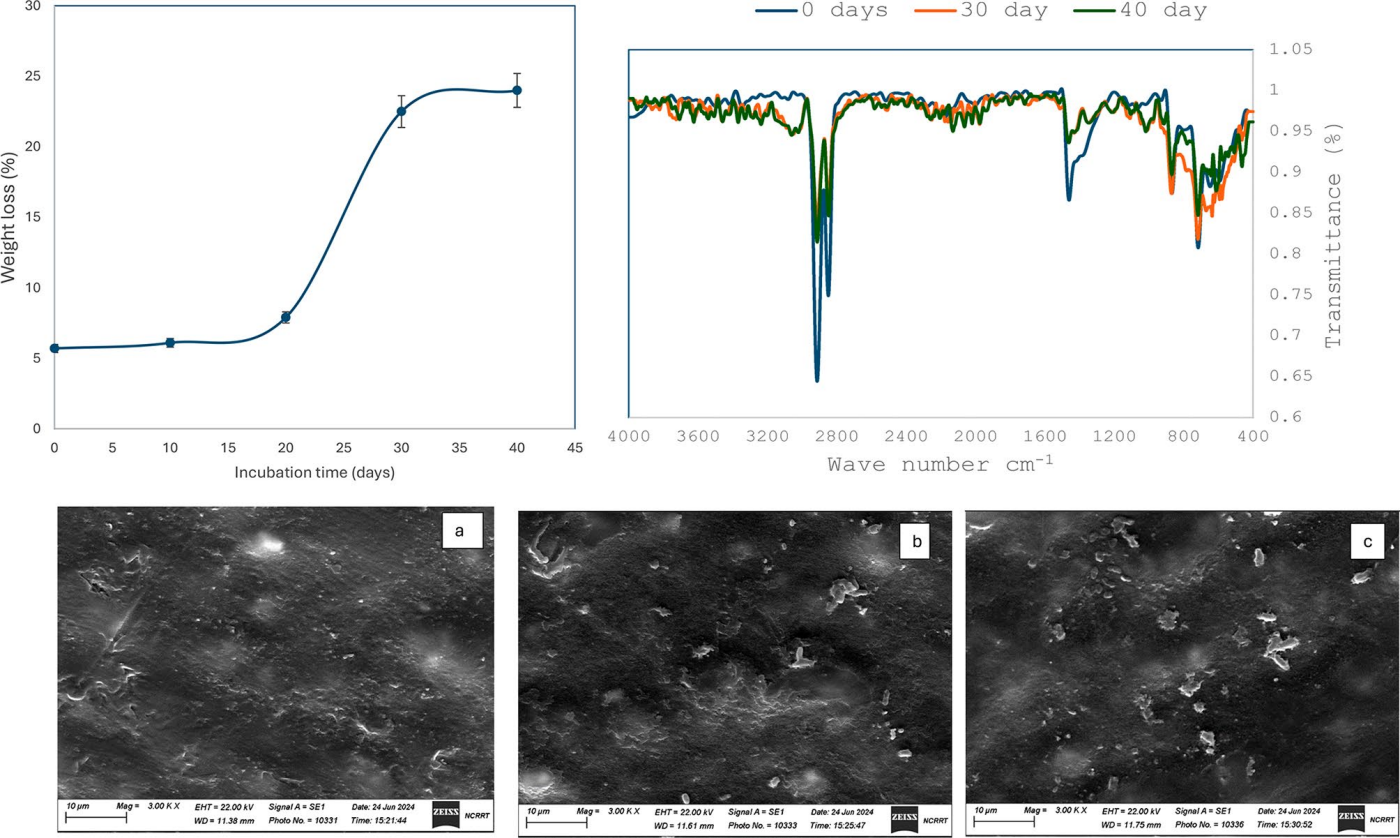
(**a**) Total weight loss for gamma irradiated LDPE MPs incubated in minimal media and 24 h biofilm formation incubated in minimal media for 0, 10, 20, 30 and 40 days. (**a**) FTIR spectra of LDPE MPs exposed to 40 kGy gamma irradiation at zero time incubation and after 30 and 40 days of incubation. (**b**) SEM images for LDPE MPs gamma irradiated and incubated at 0 (**a**), 30 (**b**) and 40 days (**c**). Magnification at 3000x

Following the pretreatment of LDPE MPs with gamma radiation and optimizing the media components to enhance colonization of the marine microbial consortium on LDPE MPs, the following step assesses the biodegradation pattern. Figure 10a shows that the increase in incubation time of MPs in supplemented media elevates the biodegradation process and results in an increase in total weight loss which increased from 5.7% for gamma irradiated LDPE MPs at zero incubation time to 22.5% after incubation for 30 days and 24% after 40 days. The obtained results show higher values in weight loss than that proved by El-Sayet et al. 2021 who reported 7% weight loss for temperature-pretreated LDPE after 112 days of incubation with *Aspergillus* strains and by Kyaw et al. 2012 who found a total weight loss of 0.3 to 20% for LDPE after 120 days using *Pseudomonas* sp. Polymer biodegradation can take place via one of two pathways: (1) hydro-biodegradation or (2) oxo-biodegradation (Nowak et al. 2011), therefore, FTIR spectrum was obtained, and carboxyl and carbonyl indices were plotted against incubation time. Figure 10a represents the FTIR spectral chart for LDPE when exposed to 40 kGy in comparison to those after gamma irradiation and biodegradation for 30 and 40 days of incubation. The spectra reveal broad peaks at 3236 cm^− 1^ and 3027 cm^− 1^ for LDPE MPs incubated for 30 and 40 days compared to a narrow one at zero incubation time These peaks indicate the presence of OH carboxylic acid, two peaks at 2902 cm^− 1^ and 28,848 cm^− 1^ which corresponds to CH characteristic for LDPE. Those transmittance peaks showed a decrease at the end of the incubation time. LDPE at zero incubation time. There was a noise evident from the range between 2133 and 1700 cm^− 1^ which indicates changes in intensities for peaks in that range. Moreover, there was a change in peak intensity at 1461 cm^− 1^ which indicates alterations in polymer structure due to microbial activity. An appearance of a separate peak at 1338 cm^− 1^ for LDPE incubated for 30 and 40 days indicates the presence of ester bond as opposed to the presence of a small hump adjoining the peak at 1461 cm^− 1^ for LDPE at zero incubation time. The obtained result indicated that the LDPE MPS under study exhibit changes at 30 days incubation with the bacterial consortium but after which, almost no structural changes take place, the data suggests that oxidation and dehydrogenation of LDPE occurred. A series of oxidation and dehydrogenation indeed were reported to influence the biodegradation of LDPE (Dey et al. 2020). The obtained SEM images for LDPE MPs (incubated for 30 and 40 days) were compared to LDPE exposed to 40 kGy gamma radiation only. The images showed morphological changes as seen in Fig. 10b. Despite rinsing LDPE MPs with alcohol and 2% SDS, the remaining bacteria were detectable after 30 and 40 days as seen in the obtained images. Such attachment can be explained by the fact that biofilms grow and mature with increased incubation time, especially in the presence of *Micrococus luteus* and *Bacillus cereus* which are two of the four identified strains in the present study and were reported to produce biosurfactant and form biofilm on LPDE. Exopolysaccharides act as sticky binding material that contains bonds able to protect the bacterial biofilm even after washing. The presence of bacteria within the plastic might also be a reason, since SEM images show structural and morphological changes, this provides a rough surface for bacteria to attach and colonize plastic surface. This result may indicate that the total weight loss of LDPE after incubation time can be more than that detected under the studied conditions. However, the overall outcome is that for efficient biodegradation of LDPE MPs, gamma irradiation could be used as a pre-treatment process, this helps bacteria to colonize LDPE MPs and form a biofilm within 24 h only. Biofilm production assists in biodegradation that takes 30 days incubation time, thus reducing the time for weight loss. One of the important aspects of plastic biodegradation is the production of by-products are considered raw materials that can be re-used for other purposes. The fungus *Phanaerocheate chrysosporium* was used to degraded PVC using lignin peroxidase and produced oxidative by-products such as carboxylic acids and alcohols (Khatoon et al. 2019). *Alternaria alternata* fungi degraded polyethylene via laccase, peroxidase enzymes and produced glycerol, organic acids and other organic compounds such as squalene (Gao et al. 2022). *Bacillus velezensis* degrades LDPE wrap with the involvement of laccase, cytochrome P450 and proprionyl-CoA carboxylase producing C24-C29 n alkanes in the media (Liu et al. 2022). Actinomycete *Gordonia polyisoprenivorans* for PE reported oxidation products (Wang et al. 2023). Yeast degradation of polyethylene terephthalate via esterase and lipase produced by-products such as benzene derivatives and alkanes (Giyahchi and Moghimi 2024). This means that the type of plastic and strain used determines the resulting biodegradation products. As far as the author’s knowledge, LDPE biodegradation using the strains in the present study have never been used before. This will be the focus of the upcoming research.

## Conclusion

LDPE Plastic bags are a common source of environmental pollution. Their exposure to environmental conditions results in their fragmentation to microplastics (MPs), which in turn present ecological and health risks. In the present study, marine consortia isolated from discarded plastics demonstrated biodegradation of LDPE MPs. Due to its hydrophobic and low water absorbent nature, a pre-treatment step using gamma irradiation was necessary to initiate the biodegradation process. This pre-treatment resulted in morphological and structural changes making them more susceptible to bacterial colonization. The addition of supplements such as TSA, biosurfactant, and CaCl_2_ to the minimal media further enhanced biofilm formation enabling bacteria to produce metabolites that would interact with LDPE MPs. Finally, incubation of 24 h pre-formed biofilm resulted in faster biodegradation than that reported in other studies. Biofilm-forming bacteria utilized LDPE MPs as a carbon source, attacking the polymer backbone via the produced metabolites (biosurfactant) or enzymes (e.g.: protease) which collectively contributed to the depolymerization of LDPE MPs. However, one major limitation for scaling-up biodegradation of plastics is the long biodegradation duration, humble weight loss percentages, and the huge quantity of plastic waste produced daily. Therefore, our future work will focus on elucidating the degradation pathways and identification of the resultant by-products to scale-up the biodegradation process in terms of quantity and quality.

## Electronic supplementary material

Below is the link to the electronic supplementary material.

Supplementary Material 1

Supplementary Material 2

## Data Availability

Data availability will be provided on request.
