# Supplementary material for: Promoting bacterial colonization and biofilm formation for enhanced biodegradation of low-density polyethylene microplastics
Source: Bioresour Bioprocess. 2025 Jun 10;12(1):59. doi: 10.1186/s40643-025-00902-8 (PMC12149076; doi:10.1186/s40643-025-00902-8)
Supplement: Supplementary file 1 — Supplementary Material 1 [file 40643_2025_902_MOESM1_ESM.docx]

Supplementary material (S1)


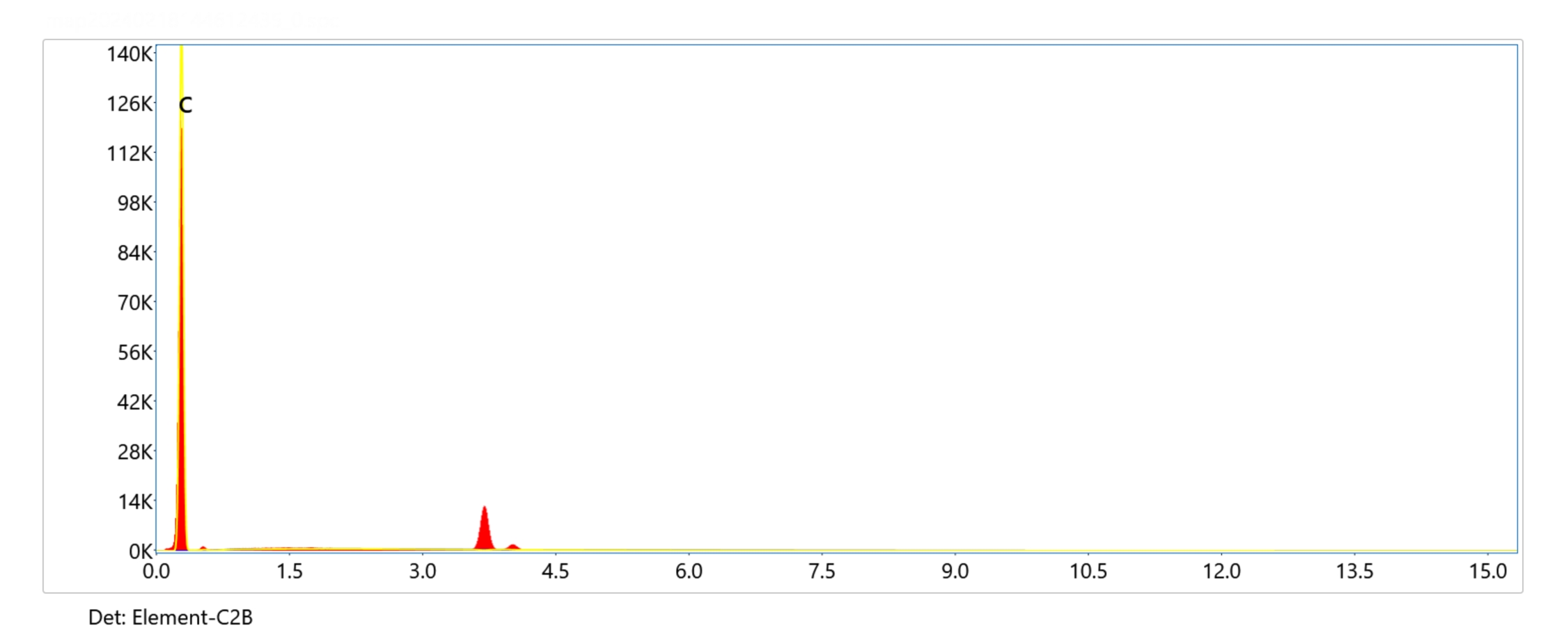


|  | Element | Weight % | Atomic % | Net Int. | Error % | R | A | F |  | |
| --- | --- | --- | --- | --- | --- | --- | --- | --- | --- | --- |
|  | C K | 100.00 | 100.00 | 5370.14 | 8.84 | 0.9439 | 0.2057 | 1.0000 |  |  |

LDPE


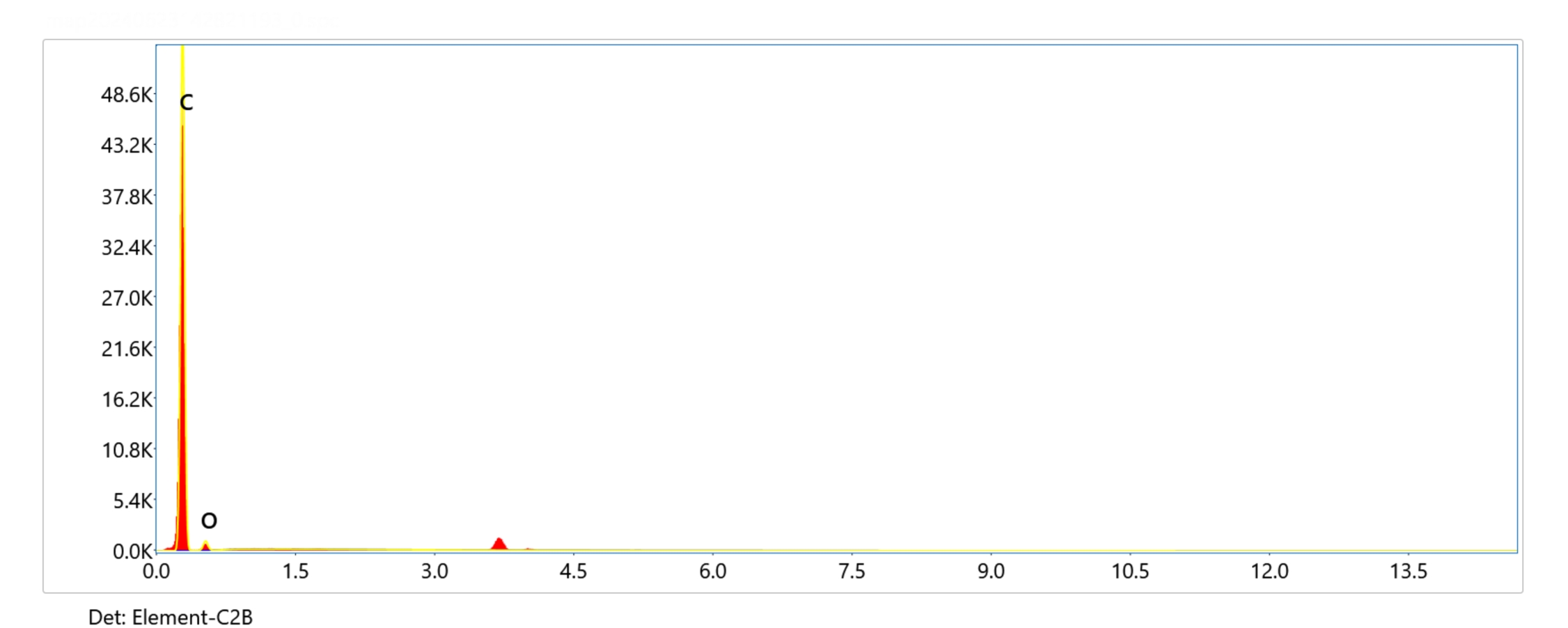


|  | Element | Weight % | Atomic % | Net Int. | Error % | R | A | F |  | |
| --- | --- | --- | --- | --- | --- | --- | --- | --- | --- | --- |
|  | C K | 93.74 | 95.22 | 2889.45 | 8.93 | 0.9429 | 0.2062 | 1.0000 |  |  |
|  | O K | 6.26 | 4.78 | 52.45 | 13.54 | 0.9500 | 0.0439 | 1.0000 |  |  |

LDPE exposed to 40 kGy gamma irradiation


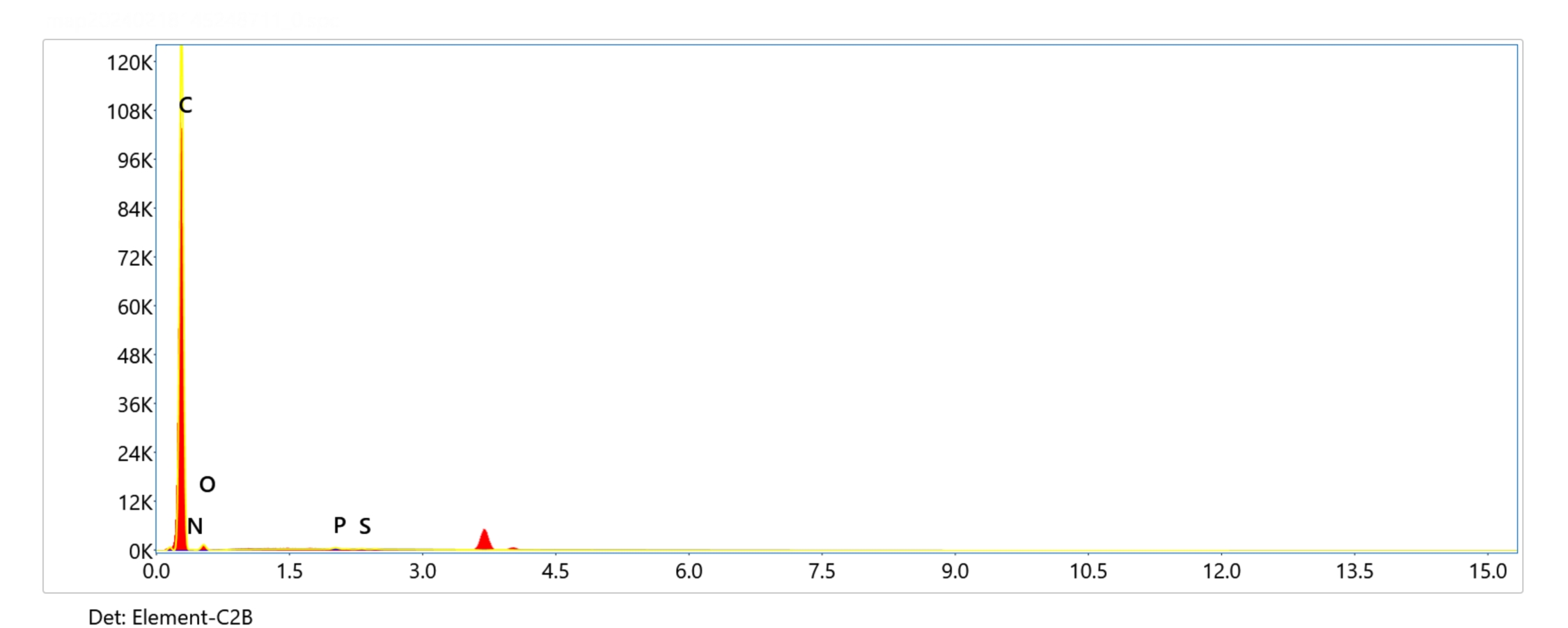


|  | Element | Weight % | Atomic % | Net Int. | Error % | R | A | F |  | |
| --- | --- | --- | --- | --- | --- | --- | --- | --- | --- | --- |
|  | C K | 95.71 | 96.74 | 5994.08 | 8.99 | 0.9431 | 0.1912 | 1.0000 |  |  |
|  | N K | 0.61 | 0.53 | 3.66 | 78.46 | 0.9469 | 0.0179 | 1.0000 |  |  |
|  | O K | 3.52 | 2.67 | 57.46 | 16.94 | 0.9500 | 0.0389 | 1.0000 |  |  |
|  | P K | 0.11 | 0.04 | 25.29 | 9.48 | 0.9662 | 0.8089 | 1.0087 |  |  |
|  | S K | 0.04 | 0.02 | 9.57 | 18.02 | 0.9680 | 0.8683 | 1.0119 |  |  |

LDPE gamma irradiated, 24 h biofilm


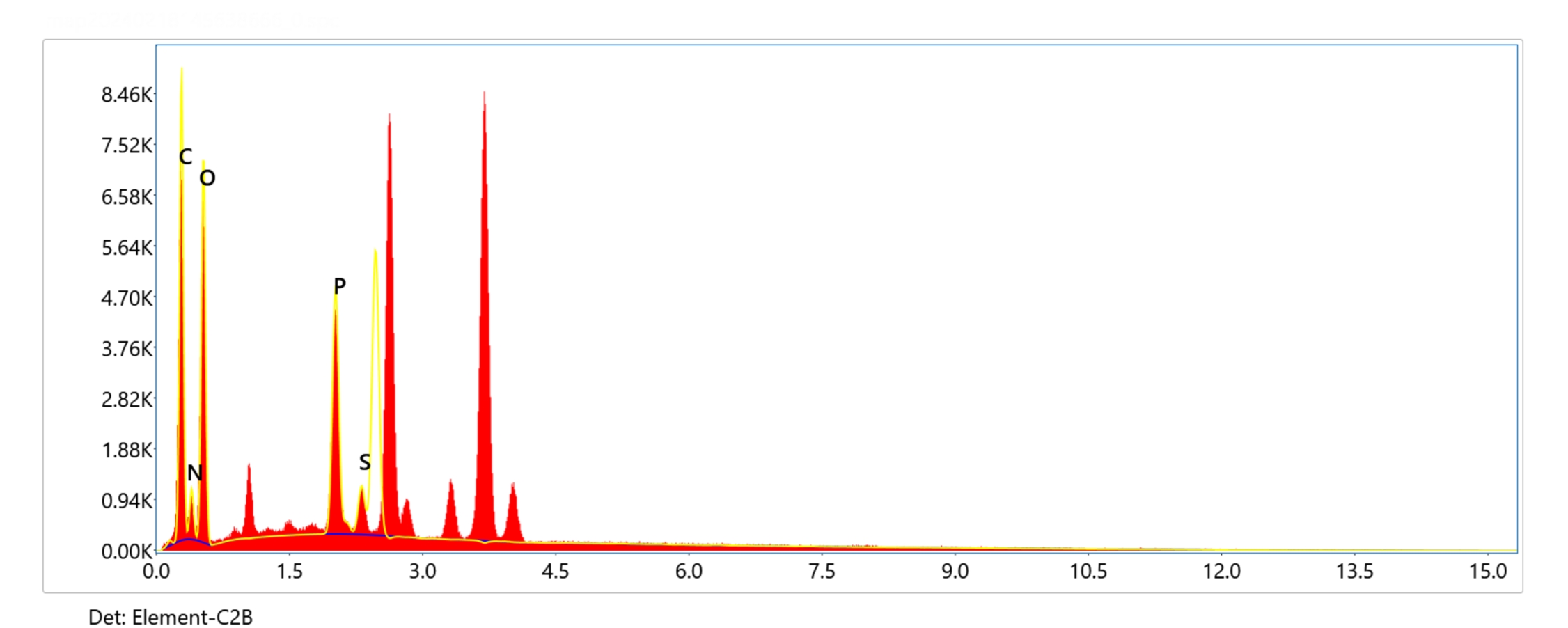


|  | Element | Weight % | Atomic % | Net Int. | Error % | R | A | F |  | |
| --- | --- | --- | --- | --- | --- | --- | --- | --- | --- | --- |
|  | C K | 43.17 | 50.85 | 540.83 | 10.55 | 0.9309 | 0.0773 | 1.0000 |  |  |
|  | N K | 10.72 | 10.82 | 61.68 | 12.79 | 0.9354 | 0.0348 | 1.0000 |  |  |
|  | O K | 40.43 | 35.74 | 505.09 | 10.71 | 0.9390 | 0.0602 | 1.0000 |  |  |
|  | P K | 4.71 | 2.15 | 476.17 | 4.30 | 0.9581 | 0.7281 | 1.0071 |  |  |
|  | S K | 0.97 | 0.43 | 99.63 | 3.78 | 0.9603 | 0.7634 | 1.0077 |  |  |

LDPE gamma irradiated, 24 he biofilm in supplemented media
