# Supplementary figures and images for: Promoting bacterial colonization and biofilm formation for enhanced biodegradation of low-density polyethylene microplastics
Source: Bioresour Bioprocess. 2025 Jun 10;12(1):59. doi: 10.1186/s40643-025-00902-8 (PMC12149076; doi:10.1186/s40643-025-00902-8)

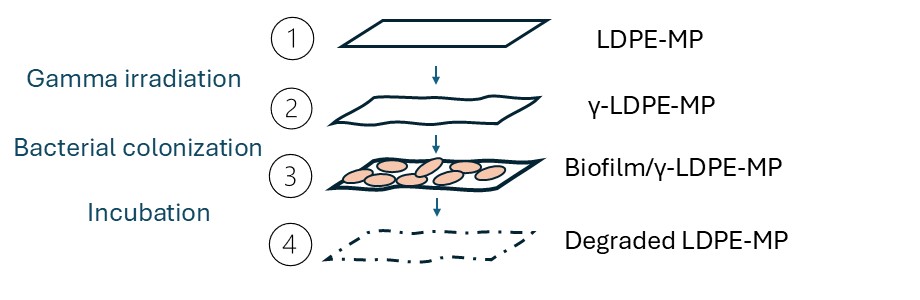

Supplement: Supplementary file 2 — Supplementary Material 2 [file 40643_2025_902_MOESM2_ESM.jpg]
